# COVID-19 Vaccine Hesitancy among Healthcare Workers and Trainees in Freetown, Sierra Leone: A Cross-Sectional Study

**DOI:** 10.3390/vaccines10050757

**Published:** 2022-05-11

**Authors:** Sahr A. Yendewa, Manal Ghazzawi, Peter B. James, Mohamed Smith, Samuel P. Massaquoi, Lawrence S. Babawo, Gibrilla F. Deen, James B. W. Russell, Mohamed Samai, Foday Sahr, Sulaiman Lakoh, Robert A. Salata, George A. Yendewa

**Affiliations:** 1 Ministry of Health and Sanitation, Freetown, Sierra Leone; syendewa@gmail.com (S.A.Y.); smidoe85@yahoo.co.uk (M.S.); drspem@gmail.com (S.P.M.); gibrilladeen1960@yahoo.com (G.F.D.); jamesbwrussell@gmail.com (J.B.W.R.); sdhsamai@yahoo.co.uk (M.S.); fsahr65@gmail.com (F.S.); lakoh2009@gmail.com (S.L.); 2 Connaught Hospital, University of Sierra Leone Teaching Hospitals Complex, Ministry of Health and Sanitation, Freetown, Sierra Leone; 3 KnowHep Foundation, Freetown, Sierra Leone; knowhepfoundation.sl@gmail.com; 4 Faculty of Health, Southern Cross University, Lismore, NSW 2480, Australia; peter.james@scu.edu.au; 5 Department of Nursing, School of Community Health Sciences, Njala University, Bo Campus, Bo, Sierra Leone; lbabawo@njala.edu.sl; 6 Department of Medicine, College of Medicine and Allied Health Sciences, University of Sierra Leone, Freetown, Sierra Leone; 7 Department of Medicine, Case Western Reserve University School of Medicine, Cleveland, OH 44106, USA; robert.salata@uhhospitals.org; 8 Division of Infectious Diseases and HIV Medicine, University Hospitals Cleveland Medical Center, Cleveland, OH 44106, USA; 9 Johns Hopkins Bloomberg School of Public Health, Baltimore, MD 21205, USA

**Keywords:** COVID-19, vaccine hesitancy, healthcare workers, Sierra Leone

## Abstract

Despite having safe and efficacious vaccines against COVID-19, vaccine hesitancy is widespread. Although a trusted source of information, vaccine hesitancy has been reported among healthcare professionals, yet few studies have explored this phenomenon in sub-Saharan Africa. We conducted a cross-sectional survey of healthcare professionals in Sierra Leone from January to March 2022. Measures included sociodemographic/health-related information and COVID-19-related concerns. From the responses, we constructed a hesitancy (VAX) score, with higher scores implying negative attitudes or unwillingness to vaccinate. Multivariate linear regression was used to access factors associated with vaccine hesitancy. Overall, 592 participants submitted responses (67.2% female, mean age 29 years, 5.6% physicians/pharmacists, 44.3% medical students, 29.2% nurses, 20.9% nursing students). The mean VAX score was 43.27 ± 8.77, with 60.1% of respondents classified as vaccine hesitant (>50th percentile) and 13.8% as highly hesitant (>75th percentile). Worries about unforeseen future effects (76.3%), a preference for natural immunity (59.5%), and profiteering/mistrust of health authorities (53.1%) were the most common concerns. Being a medical student (β = 0.105, *p* = 0.011) and previously refusing a recommended vaccine (β = 0.177, *p* < 0.001) were predictors of COVID-19 vaccine hesitancy. Our findings call for addressing vaccine hesitancy among healthcare professionals as an essential component of strategies aimed at increasing COVID-19 vaccine uptake in this setting.

## 1. Introduction

Since its emergence in 2019, the COVID-19 pandemic, caused by the novel coronavirus SARS-CoV-2, has resulted in over 450 million confirmed cases and 6 million deaths globally [1]. Population-wide lockdowns, social distancing, hand hygiene, and mask-wearing were the initial steps taken to control the pandemic [2]. Vaccination is one of the most potent and cost-effective public health tools used in preventing communicable diseases. Accordingly, the COVID-19 pandemic has led to the accelerated development of several vaccines that have proven to be safe and effective against SARS-CoV-2 [3,4,5]. Health authorities recommend vaccinating most people against COVID-19 and have identified people aged ≥ 65 years, immunocompromised hosts, e.g., people with HIV (PWH), individuals with chronic morbidities and healthcare workers (HCWs) as priority populations for vaccination [6].

Despite their proven safety and efficacy, vaccine hesitancy is posing a substantial threat to efforts seeking to combat the COVID-19 pandemic. Vaccine hesitancy has been defined as a *delay in acceptance or refusal of vaccination despite availability of vaccination services* [7]. According to the World Health Organization (WHO), vaccine hesitancy has been increasing in the last decade and was one of the top ten global health threats in 2019 [8]. Studies from the United States and other high-income countries (HICs) have reported COVID-19 vaccine hesitancy rates of >30% in the general population [9,10,11]. Vaccine hesitancy has been reported among HCWs and people who believe in the importance of vaccinations [12,13,14]. Racial minority background, poverty, low educational status, lack of information and especially misinformation, and fear and mistrust of the authorities have been identified as predictors of unwillingness to be vaccinated [12,13,14]. Negative attitudes towards vaccination result in low vaccine uptake and are exacerbating longstanding health disparities that already disproportionately impact minority communities in developed countries [12,13,14,15].

There is a paucity of studies that have explored COVID-19 vaccine hesitancy in the general population in the sub-Saharan African (SSA) context. In one of the earliest studies, Acre et al. [16] reported that willingness to vaccinate against COVID-19 was higher (67% to 88%) in five low- and middle-income countries (LMIC) in SSA (i.e., Burkina Faso, Mozambique, Nigeria, Rwanda, Sierra Leone, and Uganda) compared with the United States (65%) and Russia (30%). Despite this, vaccination rates have remained low across SSA. The Africa Centers for Disease Control and Prevention (Africa CDC) estimates that to date, about 16% and 21% of African populations have been fully or partially vaccinated, respectively [17]. One explanation for this discrepancy lies in the widespread perception that the impact of COVID-19 on SSA has not been as devastating, with the region reporting <2.5% of the global COVID-19 burden [1]. The COVID-19 policy conversation in SSA has, therefore, largely centered around equitable vaccine access, efficient supply chains, logistical and structural constraints, and funding [18]. These complexities should be taken into account in trying to understand the determinants of vaccine hesitancy in SSA.

Sierra Leone is a West African country with a population of 8 million people [19]. The country has recent experience with major public health challenges and was an epicenter of the West African Ebola epidemic of 2014–2016 [20]. The first confirmed cases of COVID-19 in Sierra Leone were reported in March 2020. Similar to other countries in the region, the Government of Sierra Leone implemented a series of population-wide lockdowns in early April through June 2020 as part of the initial virus control measures [21,22]. Although the incidence rate of COVID-19 has been reported as low (i.e., 80 cases per 100,000) [17], a recent population serosurvey estimated the national SARS-CoV-2 antibody prevalence at 2.6%, which is 43-fold higher than the number of confirmed COVID-19 cases to date [23]. While willingness to receive the vaccine has been reported as high (i.e., >80%) [16], about 30% of the population has been vaccinated [24].

As COVID-19 vaccination rollouts accelerate in SSA, establishing the determinants of vaccine hesitancy is essential to help inform an evidence-based public health policy response. Frontline HCWs and trainees interact directly with the public and are trusted sources of information yet may harbor negative attitudes towards vaccination despite being at increased risk of COVID-19 exposure [25]. In this study, we aimed to assess the prevalence and associated factors of COVID-19 vaccine hesitancy among HCWs and trainees in Sierra Leone.

## 2. Materials and Methods

### 2.1. Study Design and Population

We used a cross-sectional design to evacuate the attitudes of HCWs (i.e., doctors, pharmacists, and nurses) and trainees (i.e., students of medicine, pharmacy, and nursing) aged ≥ 18 years in Freetown, Sierra Leone, from January to March 2022.

### 2.2. Study Settings and Recruitment

Participants were enrolled at the following facilities in Freetown: (1) Faculty of Nursing, College of Medicine and Allied Health Sciences (COMAHS), (2) Connaught Hospital, (3) Ola During Children’s Hospital (ODCH) and (4) Princess Christian Maternity Hospital (PCMH). All of the HCWs and trainees were eligible for inclusion in the study. We employed a convenience sampling method to recruit HCWs and medical trainees for our study. The target population was approached by research staff at these facilities and informed about the purpose of the study. Those who consented to participation were enrolled sequentially.

COMAHS is a constituent college of the University of Sierra Leone and the country’s only medical school. It is divided into the faculties of (1) Basic Medical Sciences, (2) Clinical Sciences, (3) Pharmaceutical Sciences, and (4) Nursing. The stated goals of the college are to produce medical doctors, pharmacists, biomedical scientists, nurses, and other allied health personnel.

Connaught Hospital is a 300-bed facility located in Freetown. It is the main referral hospital for medical and surgical cases in Sierra Leone and is a major teaching affiliate of COMAHS. ODCH and PCMH are clinical affiliates of COMAHS and provide referral services for children and women, respectively.

### 2.3. Sample Size and Justification

We used the formula according to Lwanga and Lemeshow [26] to estimate the sample size, n as follows:

n = Z^2^ × p (1 − p)/e^2^

where Z = 1.96 at 95% confidence interval (CI), p = prevalence of COVID-19 vaccine hesitancy in Sierra Leone and e is the error rate. Using a COVID-19 vaccine hesitancy rate of about 20% according to a study by Acre et al. [16] and e = 5%, a minimum sample size of 245 will achieve 80% power in detecting associations between variables, assuming no association under the null hypothesis using a 2-tailed test with a significance level of 0.05.

### 2.4. Study Tools, Procedures and Measures

The survey instrument was composed of two sections. The first section entailed a questionnaire on sociodemographic and health-related information, including personal history and experience with COVID-19, routine vaccination history and general attitudes towards health, health-seeking behaviors, and trust in public health authorities.

The second section entailed a 12-item Vaccination Attitudes Examination (VAX) Scale adapted from Martin et al. [27], which has been validated within and outside of Africa [28,29,30,31]. The VAX Scale is used to evaluate general attitudes toward vaccinations across four domains: (1) mistrust of vaccine benefits, (2) worries about unforeseen future effects, (3) concerns about commercial profiteering, and (4) preference for natural immunity [27]. Responses to each of the 12 items were rated on a six-point Likert scale as follows: 1 = strongly agree, 2 = agree, 3 = slightly agree, 4 = slightly disagree, 5 = disagree and 6 = strongly disagree. To ensure clarity of questions, the survey instrument was piloted to the target study population (*n* = 20) who were not included in the study. The reliability and internal consistency of the responses were assessed using Cronbach’s alpha coefficients (α), with an overall α > 0.7 regarded as acceptable.

To estimate the prevalence of COVID-19 vaccine hesitancy, VAX scores were constructed by summing the responses of participants to items on the VAX Scale. Items 1–3 on the VAX Scale by Martin et al. [27] are positively-worded, while Items 4–12 are negatively worded. To scale all the responses in a positive direction, we reverse-scored responses to Items 4–12, with higher VAX scores implying negative attitudes or perceptions towards COVID-19 vaccination. The possible overall VAX scores range from 12 (positive attitude) to 72 (negative attitude). A respondent was defined as vaccine hesitant if their VAX score > mean or median (i.e., 50th percentile), assuming a normal distribution of VAX scores. Similar to Oke et al. and others [32,33], vaccine hesitancy was further stratified as low-level when the VAX score was between the 25th and 50th percentiles (i.e., 12–32), moderate when the VAX score was between the 50th and 75th percentiles (i.e., 33–52), and high when the VAX score was greater than the 75th percentile (i.e., >52).

### 2.5. Statistical Analysis

Statistical analyses were performed using the SPSS Version 28.0 (Armonk, NY, USA; IBM Corp). Categorical variables were reported as frequencies (percentages) and continuous variables as means (standard deviation) or medians (range). Multivariable linear regression was used to identify factors associated with COVID-19 vaccine hesitancy, represented by the variable VAX scores. Covariates with *p* < 0.05 in the univariate analysis were included in the multivariate model, with statistical significance set at *p* < 0.05.

### 2.6. Ethical Approval

Ethical approval was obtained from the Sierra Leone Ethics and Scientific Review Committee (approval date 20 December 2021). Prior to enrolment, it was explained to participants that providing verbal consent and answering the survey questions implied informed consent. Participation was strictly voluntary, and the participants could withdraw from the study at any stage.

## 3. Results

### 3.1. Sample Description

Table 1 describes the socio-demographic characteristics of the survey participants.

We received responses from 592 survey participants. The majority were female (67.2%), mean age 29.03 ± 6.81 years, single (72.6%), with the highest contribution from medical students (44.3%). Furthermore, 27.7% had been tested for COVID-19, and the self-reported COVID-19 positivity rate was 3.3%. About 38.3% had received a COVID-19 vaccine, while 29.7% had received a hepatitis B vaccine, and 7.9% had received an Ebola vaccine. Additionally, 14.0% had previously refused a recommended vaccine.

### 3.2. Attitudes towards Vaccines

The respondents’ attitudes towards COVID-19 vaccination are presented in Table 2. Reliability and internal validity were demonstrated, with α ranging from 0.67 to 0.82 across domains (subscales) and 0.76 overall.

### 3.3. Vaccine Hesitancy Scores and Distribution

**Figure 1:**
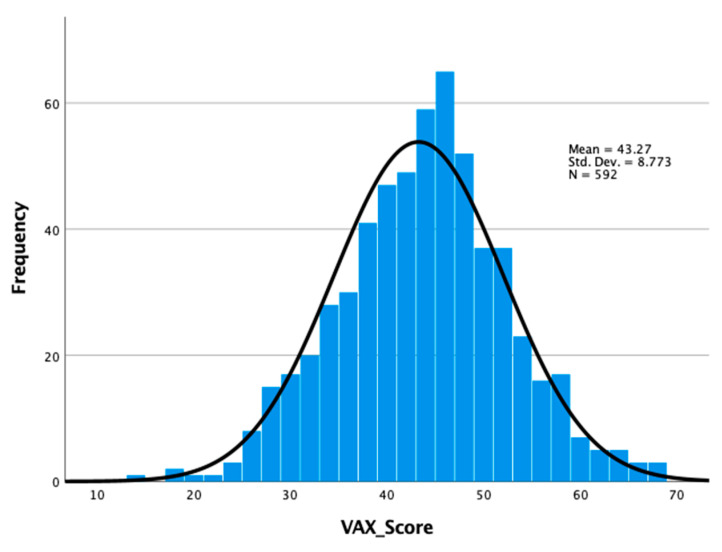
Distribution of VAX Scores.

Overall and subscale (domain) VAX scores are displayed in Table 3. The VAX scores were normally distributed (Figure 1) and ranged from 14 to 68, with an overall hesitancy mean VAX score of 43.27 ± 8.77, indicating that about 60.1% of survey respondents were hesitant and unlikely to vaccinate against COVID-19. Furthermore, 11.5%, 74.7%, and 13.8% were classified in the low-, mild-to-moderate- and high-level vaccine hesitancy categories, respectively.

Across subscales (domains), worries about unforeseen future effects (76.3%) and preference for natural immunity (59.5%) were the most common reasons for concerns around COVID-19 vaccination, followed by concerns about commercial profiteering.

### 3.4. Factors Associated with COVID-19 Vaccine Hesitancy

Table 4 summarizes the factors associated with greater COVID-19 vaccine hesitancy. The univariate and multivariate linear regression showed that being a medical student (β = 0.105, *p* = 0.011) and having previously refused a recommended vaccine (β = 0.177, *p* < 0.001) were associated with greater COVID-19 vaccine hesitancy.

## 4. Discussion

In this study, we assessed COVID-19 vaccine hesitancy and associated factors among a large and diverse group of HCWs (physicians, pharmacists, nurses) and trainees (medical and nursing students) in Freetown, Sierra Leone. About 60.1% of the survey respondents were classified as COVID-19 vaccine hesitant, while 13.8% were classified as highly hesitant and therefore unlikely to vaccinate. In comparison, 38.3% of the survey respondents self-reported having already received a COVID-19 vaccine. Interestingly, the proportion of HCWs who have already received a COVID-19 vaccine (i.e., 38.3%) and those classified as vaccine hesitant in our study (i.e., 60.1%) add up to approximately 100%, suggesting that barriers other than equitable vaccine access could be the main determinants of COVID-19 vaccine hesitancy among HCWs in this setting.

Overall, the COVID-19 vaccine hesitancy levels in our study were higher than those that have been reported in studies conducted among HCWs in HICs (i.e., France, United States, Qatar) [31,34,35,36] but lower than findings reported in studies from Africa (i.e., Ethiopia, Nigeria, and South Africa) [28,37,38,39]. The difference in COVID-19 vaccine hesitancy levels between our study and previous African studies may be due to how COVID-19 vaccine hesitancy was measured, study populations and differences in study periods, as well as the impact the COVID-19 pandemic has had on the study populations.

The high levels of COVID-19 vaccine hesitancy reported among HCWs in our study are cause for concern, given that our study was conducted at a time when scientific evidence for various COVID-19 vaccines had already been made widely available. This suggests that the availability of evidence in support of the efficacy and safety of vaccines in itself may not be enough to overcome apprehension toward COVID-19 vaccination. Instead, socio-cultural factors may strongly influence vaccine uptake decisions. In addition, the high levels of COVID-19 vaccine hesitancy observed in our study could cause an erosion of public confidence and trust, which may, in turn, lead to negative repercussions for vaccine uptake in the general public, given that HCWs are considered role models and a trustworthy source of health information.

In line with previous studies [37,38], worries about unforeseen future effects and a preference for natural immunity were proffered as the primary causes for concerns around COVID-19 vaccination among survey respondents. Although not captured in our study, the effect of mixed-messaging regarding COVID-19 vaccination, especially on social media, platforms might explain HCWs’ concerns. The effect of social media on COVID-19 vaccine uptake decision in Africa has been documented [37,38]. This underscores the critical need for an effective information communication strategy to dispel misconceptions around vaccine efficacy and safety.

A noteworthy finding of our study was that COVID-19 vaccine hesitancy was associated with being a medical student. The reasons for this association are unclear and warrant further investigation. COVID-19 vaccine hesitancy has been reported in studies conducted specifically among medical students in both HICs and LMICs. In one report from the United States by Lucia et al. [40], 23% of medical students surveyed were unwilling to take a COVID-19 vaccine even after approval by the Food and Drug Administration. In comparison, studies conducted among Sudanese, Egyptian, Nigerian and Ugandan medical students have generally shown higher COVID-19 vaccine hesitancy rates ranging from 30% to 60% [41,42,43,44]. Concerns around vaccine safety and efficacy, trust in health authorities, healthcare costs, and experience in the healthcare field were some of the major drivers of vaccine hesitancy in these studies [41,42,43,44]. These factors should be investigated in future studies using qualitative methods to better understand the correlates of COVID-19 hesitancy among medical students in Sierra Leone.

Studies from HICs have shown that general attitudes towards essential childhood immunization and routine vaccines such as the seasonal influenza vaccine can predict the willingness to vaccinate against COVID-19 [27,45,46,47]. We tested this hypothesis in our study using HCWs’ hepatitis B and Ebola vaccination status to fit the local Sierra Leonean context. Hepatitis B immunization is recommended for all adults, including HCWs [48]; we, therefore, used hepatitis B vaccination status as a marker for compliance with required routine vaccinations. Ebola vaccination status was tested as a surrogate for HCWs’ attitudes towards novel or emerging communicable diseases, given the recent history of an Ebola epidemic in the country [49]. We found that although there was a trend towards lower COVID-19 vaccine hesitancy among HCWs who had previously received the hepatitis B or Ebola vaccines, this association did not attain statistical significance. This could be partly explained by the observation that in many LMICs, coverage for most routine vaccines is generally low and rarely enforced [50]. In our study, only 29.7% and 7.9% of HCWs had received a hepatitis B or Ebola vaccine, respectively.

Similarly, we also observed that survey respondents were more likely to be COVID-19 vaccine hesitant if they had previously refused a recommended vaccine. Negative experiences, especially serious adverse reactions to previous vaccines, can shape attitudes towards vaccination and may help explain this association. This is supported by the fact that worries about unforeseen future effects were the most commonly reported concern regarding COVID-19 uptake among HCWs in our study.

Our study had a few limitations worthy of discussion. Firstly, our survey employed convenience sampling, which may have resulted in an underestimate of the true prevalence of COVID-19 vaccine hesitancy. Secondly, the study was restricted to Freetown, an urban setting, and may not reflect HCWs’ attitudes towards COVID-19 vaccination nationally. Thirdly, our exploration of barriers to vaccine uptake was not exhaustive, which may be better assessed using a qualitative or mixed-methods study design. Nonetheless, our findings contribute to our understanding of COVID-19 hesitancy among HCWs in Sierra Leone and could inform interventions aimed at increasing COVID-19 vaccine uptake in this population.

## 5. Conclusions

A high prevalence of COVID-19 vaccine hesitancy was observed among healthcare professionals in Freetown, Sierra Leone. Worries about unforeseen future effects, a preference for natural immunity, and profiteering/mistrust of health authorities were the most common concerns expressed. Being a medical student and previously refusing a recommended vaccine were strong determinants of COVID-19 vaccine hesitancy. Given that healthcare professionals are disproportionately at risk of COVID-19 exposure and are a trusted source of information for the general public, our findings call for addressing vaccine hesitancy among this group as an essential component of the overall strategies aimed at increasing COVID-19 vaccine uptake in this setting.

## Figures and Tables

**Table 1:** Sociodemographic and health characteristics of participants (N = 592).

Variables	N	%
**Gender**		
Male	194	32.8
Female	398	67.2
**Age, years**		
Mean ± SD	29.03 ± 6.81	
<25	182	30.7
25–34	286	48.3
35–44	104	17.6
≥45	20	3.3
**Healthcare worker or trainee**		
Physician/Pharmacist	33	5.6
Medical student	262	44.3
Registered nurse	173	29.2
Nursing student	124	20.9
**Relationship status**		
Single	430	72.6
Married	144	24.3
Undeclared	18	3.0
**Health-related questions**		
Ever been tested for COVID-19	164	27.7
Self-reported COVID-19 positivity rate	19	3.3
Treated or care for patient with COVID-19	80	13.5
Family member tested positive for COVID-19	40	6.8
Received COVID-19 vaccine	227	38.3
Received Hepatitis B vaccine	176	29.7
Received an Ebola vaccine	41	6.9
Ever refused a recommended vaccine	83	14.0
History of chronic illness	38	6.4

Abbreviations: SD, standard deviation.

**Table 2:** Summary of COVID-19 attitude statements and vaccine hesitancy levels (in percentages, %).

Attitude Statement	Low/Mild	Moderate	High	Domain Cronbach’s Alpha
**Mistrust of vaccine benefits**				
I feel that the COVID-19 vaccine is very safe I can rely on the COVID-19 vaccine to prevent serious infection with COVID-19 I feel fully protected from COVID-19 infection in the future after getting the COVID-19 vaccine	42.5	43.4	14.1	0.82
	40.5	37.6	21.9	
	30.2	44.9	24.9	
**Worries about unforeseen future effects**				
4. Although the COVID-19 vaccine appears to be safe, there may be problems with the vaccine that we have not yet discovered I (r) 5. The COVID-19 vaccine can cause unforeseen problems in the future(r) 6. I worry about the unknown future effects of the COVID-19 vaccine (r)	6.5	17.7	75.8	0.73
	18.4	38.4	43.2	
	8.7	19.1	72.2	
**Concerns about commercial profiteering**				
7. COVID-19 vaccine will make a lot of money for pharmaceutical companies but will not bring much benefit to common people (r) 8. Authorities promote the COVID-19 vaccine for financial gain, not for people’s health (r) 9. COVID-19 vaccination programs are a fraud (r)	30.2	29.3	40.5	0.78
	44.6	30.4	25.0	
	59.8	27.7	12.5	
**Preference for natural immunity**				
10. Natural immunity will last longer than immunity from the COVID-19 vaccine (r) 11. Natural exposure to the virus gives the safest protection against COVID-19 (r) 12. Being exposed to COVID-19 naturally is safer for the immune system than being exposed through vaccination (r)	11.3	25.5	63.2	0.67
	47.3	29.6	23.1	
	45.3	30.7	24.0	

Abbreviations: (r), reverse-scored in a positive direction on a six-point Likert scale ranging from 1 (strongly disagree) to 6 (strongly agree) and reclassified hesitancy as low (1 and 2), moderate (3 and 4) and high (5 and 6).

**Table 3:** Domain and Overall Vaccine Hesitancy Scores.

Variables	Expected Range of VAX Score	VAX Score or N	% Highest Possible VAX Score
**Overall hesitancy**			
Mean (SD)	12–72	43.27 ± 8.77	60.1
Median (Min-Max)	12–72	44 (14–68)	61.1
**Hesitancy domains**			
Mistrust of vaccine benefits	3–18	9.25 ± 3.49	51.4
Worries about unforeseen future effects	3–18	13.73 ± 2.93	76.3
Concerns about commercial profiteering	3–18	9.56 ± 3.72	53.1
Preference for natural immunity	3–18	10.73 ± 3.18	59.5
**Level of hesitancy**			
Low	12–32	68	11.5
Mild to moderate	33–52	442	74.7
High	>52	82	13.8

Abbreviations: N, sample size; Min, minimum; Max, maximum; SD, standard deviation; VAX Score, vaccine hesitancy score.

**Table 4:** Univariate and multivariable linear regression correlates of COVID-19 vaccine hesitancy.

Variables	Univariate			Multivariable		
	β	S.E.	*p*-Value	β	S.E.	*p*-Value
**Sociodemographic information**						
Sex: male	0.043	0.768	0.291			
Age (years)	−0.030	0.053	0.468			
Relationship status: single	0.006	0.809	0.887			
**Healthcare worker or trainee**						
Physician/Pharmacist	−0.060	0.371	0.142			
Medical student	0.115	0.722	0.005	0.105	0.730	0.011
Registered nurse	−0.070	0.792	0.088			
Nursing student	−0.028	0.886	0.503			
**Health information**						
Ever tested for COVID-19	−0.052	0.805	0.205			
Tested positive for COVID-19	0.034	2.046	0.412			
Treated or cared for patient with COVID-19	0.025	1.055	0.542			
Family member tested positive for COVID-19	0.022	1.437	0.585			
Received Hepatitis B vaccine	−0.044	0.789	0.281			
Received Ebola vaccine	−0.088	1.416	0.032	−0.061	1.427	0.139
Refused a recommended vaccine in the past	0.175	1.023	<0.001	0.177	1.016	<0.001
History of any chronic illness	0.048	1.471	0.246			

## Data Availability

The data presented in this study are available on request from the corresponding author.
